# Roadmap for mainstreaming integrated pest management (IPM) into a climate smart one-health (CS-OH) framework

**DOI:** 10.1016/j.onehlt.2025.101084

**Published:** 2025-05-21

**Authors:** Henri E.Z. Tonnang, Ghislain T. Tepa-Yotto, Bonoukpoè Mawuko Sokame, Jeannette K. Winsou, Manuele Tamò, Rousseau F. Djouaka

**Affiliations:** a International Centre of Insect Physiology and Ecology (*icipe*), P.O. Box 30772-00100, Nairobi, Kenya; b University of KwaZulu-Natal, School of Agricultural, Earth, and Environmental Sciences, Pietermaritzburg 3209, South Africa; c Biorisk Management Facility (BIMAF), International Institute of Tropical Agriculture (IITA-Benin), 08 BP 0932 Tri Postal, Cotonou, Benin; d Ecole de Gestion et de Production Végétale et Semencière (EGPVS), Université Nationale d'Agriculture (UNA), BP 43 Kétou, Benin; e International Institute of Tropical Agriculture (IITA-Ghana), Accra, Ghana

**Keywords:** Digital twin, Interconnectedness, System thinking, System dynamics, Multi-disciplinary

## Abstract

Climate change presents significant challenges to agricultural sustainability, particularly in integrated pest management (IPM). To address these challenges, we propose a holistic and interdisciplinary conceptual framework, climate-smart One-Health (CS-OH), which integrates ecological, environmental, and socio-economic factors, considering human, animal, soil, and water health within the environment. This paper introduces a roadmap for Climate-Smart OH IPM, combining One-Health (OH) principles with climate-smart agriculture to promote sustainable pest management amid climate change. The roadmap utilizes Systems Thinking (ST) & System Dynamics (SD) methodologies to comprehend complex interactions in climate-affected agricultural systems. Additionally, we provide a step-by-step implementation of Digital Twin (DT) IPM, employing virtual models and real-time data for dynamic pest management. The roadmap's significance lies in optimizing resource allocation, fostering biodiversity, mitigating climate impact, and preventing zoonotic diseases. Furthermore, promoting adaptive pest management practices enhances agricultural system resilience. Through adopting this transformative roadmap, stakeholders can actively contribute to sustainable pest management and foster a healthier world for all.

## Highlights

• Proposing CS-OH that combines ecological, environmental, and socio-economic factors, addressing human, animal, soil, and water health.
• Integrating One-Health principles with climate-smart agriculture for sustainable pest management amid climate change.
• Employing Systems Thinking (ST) & System Dynamics (SD) to understand complex interactions in climate-affected agriculture.
• Introducing Digital Twin (DT) IPM with virtual models and real-time data for adaptable pest control.
• Roadmap optimizes resources, biodiversity, climate impact, disease prevention, and enhances agricultural resilience.

## 1 Introduction

### 1.1 Climate change and its impact on ecosystems

Climate change has become a pervasive challenge with profound implications for ecosystems, agriculture, and food security. The rise in global temperatures and alterations in precipitation patterns are disrupting ecological balances, including the dynamics of pest populations. These changes have underscored the need for innovative pest management strategies that are adaptive, sustainable, and resilient to the impacts of climate change. Integrated Pest Management (IPM) offers a promising pathway to address these challenges by employing a diverse array of pest control strategies that minimize reliance on chemical pesticides while safeguarding the environment. A notable example of climate-smart biological control is the use of *Amblyseius swirskii*, a predatory mite, for managing whiteflies and thrips in crops like tomatoes and cucumbers. This method not only reduces the need for chemical pesticides but also enhances ecological balance, as the mites thrive under varying climatic conditions, making them an adaptive and sustainable solution [1,2].

In the realm of mechanical controls, pheromone traps have gained traction as a climate-smart IPM tool. These traps, which are species-specific and environmentally benign, have been successfully used in managing pests like the fall armyworm (*Spodoptera frugiperda*), a major threat to maize production. The use and application of these methods, f can help farmers to adapt to pest outbreaks driven by climate variability without increasing their environmental footprint [[3], [4], [5], [6]]. These changes pose significant risks to agriculture, forestry, and natural ecosystems, affecting crop yields, forest health, and biodiversity [7].

One Health (OH) has gained considerable prominence since the beginning of the 21st century, driven by recent epidemics and the increasing importance of zoonotic diseases [[8], [9], [10], [11]]. However, the focus has primarily been on the interactions between animal and human health, with less attention given to environmental and plant health. Nevertheless, there is growing evidence that addressing the challenges of climate change, food and nutritional insecurity, and biodiversity loss is best accomplished within the framework of One Health. Integrated Pest Management (IPM), a concept that encompasses multiple pest control strategies, can be integrated into the One Health approach [12]. Historically, the application of IPM has demonstrated its multifaceted nature. For instance, biological control agents such as parasitoids and predators have been employed alongside habitat management techniques like intercropping and the use of cover crops to enhance ecosystem services. These practices are often coupled with cultural adjustments, such as altering planting dates to disrupt pest life cycles, and mechanical methods like pheromone traps to monitor and reduce pest populations. Chemical controls, when used, are carefully integrated as a last resort, targeting specific pests with minimal non-target effects. Examples such as the push-pull strategy for controlling stemborers in maize fields [3], and the use of natural predators like *Trichogramma* wasps to manage caterpillar pests illustrate the inherently collaborative and adaptive framework of IPM. The strength of IPM lies in its dynamic, systems-based approach, which not only addresses the immediate challenges of pest control but also aligns with broader goals such as biodiversity conservation, soil health, and climate resilience. Therefore, this manuscript builds upon IPM's foundational principles by proposing its integration into the Climate-Smart One Health (CS-OH) framework, emphasizing adaptability to climate variability and a focus on interconnected human, animal, plant, and environmental health. This integrated perspective provides a path forward for enhancing IPM's role in addressing the complex challenges posed by climate change and increasing agricultural sustainability [13,14].

### 1.2 The concept of One Health (OH) Integrated Pest Management (IPM)

The concept of One Health (OH) emphasizes the interconnectedness of human health, animal health, and the environment, recognizing that the health of humans, animals, and ecosystems are intimately linked [15]. OH, promotes collaborative and interdisciplinary approaches to address complex health challenges, including those related to integrated pest management (IPM). IPM is a holistic approach to pest management that integrates various strategies and tools to minimize the use of chemical pesticides and mitigate environmental impacts while effectively managing pest populations. OH, complements IPM by considering the broader ecological and health implications of pest management practices [5]. The relevance of OH IPM lies in its recognition of the interactions between pests, hosts, and the environment. Pest infestations can have significant implications for human and animal health, as well as ecosystem integrity. For example, certain pests can transmit diseases to humans and animals, such as mosquitoes transmitting vector-borne diseases like malaria or ticks transmitting Lyme disease. The adoption of OH IPM approach is not solely on pest eradication but also on minimizing the risks associated with pests and their management strategies. This includes considering the potential impacts of pest control methods on non-target organisms, including beneficial insects, wildlife, and water resources [16]. Additionally, the use of chemical pesticides can lead to the development of pesticide resistance in pests, posing long-term challenges for effective pest management [5]. Implementing OH IPM principles involves collaboration among various stakeholders, including farmers, veterinarians, entomologists, ecologists, public health officials, and policymakers. These stakeholders work together to identify sustainable and integrated solutions that balance pest control efficacy with environmental and health considerations. For example, a OH approach may involve using biological control methods, such as the introduction of natural predators or pathogens, to manage pest populations rather than relying solely on chemical pesticides. This approach minimizes the risks associated with chemical residues and fosters a more balanced ecosystem. Furthermore, monitoring and surveillance systems that integrate data on pest dynamics, disease prevalence, climate conditions, and ecosystem health can provide early warning signals for potential outbreaks and help guide targeted interventions [5]. By considering both human and animal health aspects, such systems contribute to effective pest control while reducing the risks of disease transmission.

### 1.3 Importance of climate-smart One Health (OH) integrated pest management (IPM)

Climate change poses significant challenges to agricultural systems, including IPM. Climate-smart approaches in IPM are essential for adapting to changing environmental conditions, minimizing pest-related risks, and promoting sustainable agricultural practices. Here, we elaborate on the importance of climate-smart approaches in IPM. Climate change influences the distribution, abundance, and behaviour of pests, which can disrupt pest management strategies. Climate-smart approaches in IPM emphasize the need to adapt pest management practices to the changing pest dynamics [17]. When we integrate climate data into decision-making processes, we can anticipate shifts in pest populations and modify control measures accordingly. Climate-smart IPM utilizes advanced technologies and predictive models to detect and predict pest outbreaks. The integration of climate data, pest monitoring information, and other relevant factors, such as crop phenology and socio-economic indicators, early warning systems can be developed to alert farmers and enable timely interventions [18]. Early detection and prediction allow for more targeted and effective pest management strategies. Climate-smart approaches in IPM optimize the use of pest control measures to minimize their environmental impact and maximize their efficacy. The consideration of climate and weather conditions, pest life cycles, and ecological interactions, farmers can determine the most appropriate timing and dosage of pest control interventions [19]. This reduces unnecessary pesticide use, minimizes resistance development, and preserves natural enemies and beneficial organisms. Climate-smart IPM promotes the resilience and adaptability of agricultural systems in the face of climate change. The adoption of diversified pest management strategies, such as crop rotation, intercropping, and agroforestry, etc., farmers can enhance the overall resilience of their systems to pest pressures [20]. Such approaches also improve the capacity of agroecosystems to withstand extreme weather events and climate variability. Climate-smart IPM requires the integration of multiple disciplines, including entomology, climatology, agronomy, and socio-economic sciences. Collaborative efforts among researchers, farmers, policymakers, and extension services are crucial for developing and implementing climate-smart IPM strategies. This interdisciplinary approach ensures that pest management practices are tailored to local contexts and are responsive to climate-related challenges. Climate-smart IPM aligns with the principles of sustainability and environmental stewardship by reducing the reliance on synthetic pesticides and minimizing the environmental footprint of pest management practices. It promotes the use of ecological approaches, such as biological control, habitat manipulation, and cultural practices, which enhance ecosystem services and reduce negative impacts on non-target organisms [5].

## 2 Materials and methods

**Fig. 1:**
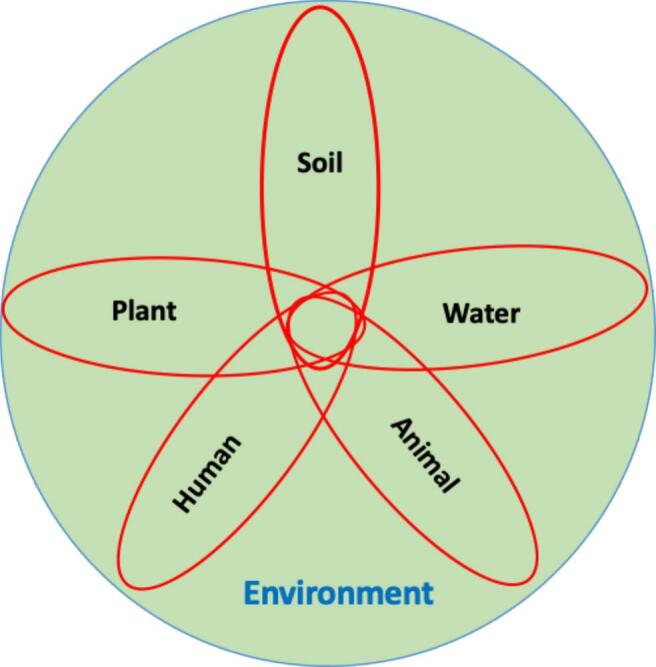
Climate-Smart OH IPM is an innovative and holistic approach that recognizes the interdependence of human, water, animal, plant, and soil health, fostering resilience in agroecosystems and promoting sustainable pest management. It integrates knowledge from diverse fields like entomology, plant pathology, climatology, climate science, public health, and veterinary science.

This study introduces a novel concept, namely, Climate-smart One-Health Integrated Pest Management (CS-OH IPM) that we find essential to define and contextualize before delving into the core of the research. Climate-smart OH IPM is a pioneering and comprehensive approach that merges the principles of OH and climate-smart agriculture to tackle pest management challenges amidst climate change. This integrated approach acknowledges the intricate interconnections among human health, animal health, plant health, soil health, and the environment (Fig. 1), while also considering the impacts of climate change on pest dynamics and agriculture.

### 2.1 Data for climate-smart OH IPM

The climate-smart OH IPM relies on diverse datasets [13] to effectively address pest management challenges in the context of climate change. Here are the different datasets needed for a comprehensive analysis of climate-smart OH IPM:

• *Climate Data:* High-resolution climate data is essential for understanding how climate change influences pest dynamics and distribution patterns. Variables such as temperature, precipitation, humidity, and extreme weather events play a crucial role in shaping arthropod populations and their interactions with the environment.
• *Arthropod Population Dynamics:* Long-term monitoring data on arthropod populations is crucial for assessing changes in their abundance, distribution, and phenology over time. This data helps identify pest outbreaks, emergence of new pest species, and potential shifts in pest-plant interactions.
• *Pest-Plant Interactions:* Information on pest-plant interactions, including host range, feeding preferences, and oviposition sites, is vital for understanding the drivers behind pest outbreaks and potential crop damage. This data informs targeted pest management strategies and the selection of appropriate pest control measures.
• *Environmental Variables:* Datasets that capture environmental factors, such as soil characteristics, land use, and habitat types, provide insights into the ecological context of pest dynamics. Understanding how environmental changes influence pest populations aids in designing context-specific and sustainable pest management interventions.
• *Disease and Health Data:* For zoonotic pests, data on disease prevalence in human and animal populations is essential to assess potential health risks and identify links between human, animal, and arthropod health.
• *Ecosystem Health Indicators*: Ecosystem health indicators, such as biodiversity metrics and ecosystem services assessments, help in evaluating the resilience and functionality of agroecosystems. Healthy ecosystems with diverse natural enemies and beneficial organisms contribute to effective pest control.
• *Climatic Projections:* Future climate projections are crucial for anticipating potential changes in pest dynamics and the effectiveness of current pest management practices. These projections inform adaptive strategies to combat emerging pest challenges under changing climatic conditions.
• *Remote Sensing Data:* Satellite imagery and remote sensing data provide a holistic view of land cover changes, vegetation health, and environmental conditions that influence pest populations and their habitats.

Collecting, analyzing, and integrating these datasets facilitate data-driven decision-making, helping to develop climate-smart OH strategies for sustainable and effective Integrated Pest Management. To establish a comprehensive roadmap for Climate-smart OH IPM, we propose two main methodological approaches including system thinking & system dynamics (ST&SD) and Digital Twin (DT).

### 2.2 Systems thinking (ST) & System dynamics (SD) for climate-smart one-health integrated pest management (OH IPM)

Step-by-step Implementation of ST & SD for Climate-Smart One-Health Integrated Pest Management (OH IPM):

*Step 1. Stakeholder Collaboration:* Engage relevant stakeholders, including farmers, researchers, policymakers, healthcare professionals, and environmental organizations, to define goals, priorities, and local contexts. Stakeholder collaboration ensures that the model reflects diverse perspectives, practical challenges, and real-world applicability.

*Step 2. System Analysis*: The second step in implementing ST & SD for Climate-Smart OH IPM is to conduct a comprehensive analysis of the agricultural system. This includes identifying the key components, relationships, and feedback loops that influence pest dynamics, human health, animal health, plant health, soil health, and the environment. Understanding the system's structure and dynamics is crucial for developing effective management strategies.

*Step 3. Identifying Variables and Relationships:* Next, the relevant variables that impact pest management, climate change, human and animal health, and ecosystem health are identified. These variables include temperature, precipitation, pest populations, crop health, disease prevalence, pesticide use, human behaviours, environmental factors. The relationships and interactions between these variables are also determined, considering both direct and indirect influences.

*Step 4. Conceptual and dynamic Modelling*: Once the variables and relationships are identified, a conceptual model is developed using ST and SD approaches. The model captures the cause-and-effect relationships, feedback loops, and time delays that influence the system's behaviour. Thereafter, the conceptual model is transformed into a dynamic model by employing stocks, flows, auxiliary links, and clouds, which enable the system to be numerically simulated and quantified. The conceptual model acts as a blueprint for understanding the complexity of the integrated pest management system and guiding subsequent analyses.

*Step 5. Data Collection and Model Calibration:* The model requires real-world data to calibrate and validate its accuracy. Data on pest populations, climate variables, health indicators, and agricultural practices are collected from various sources, including field surveys, weather stations, health records, and agricultural databases. The model is calibrated to fit observed data and is validated to ensure its reliability in replicating real-world dynamics.

*Step 6. Scenario Analysis:* Once the model is calibrated and validated, scenario analysis is conducted to explore different pest management and health intervention strategies. Various scenarios are simulated to assess their potential impacts on pest dynamics, human and animal health, and ecosystem health under different climate change conditions. This analysis helps identify effective strategies for climate-smart integrated pest management.

*Step 7. Policy Formulation and Decision Support:* The insights gained from the scenario analysis guide the formulation of policies and strategies for climate-smart One-Health Integrated Pest Management (IPM). Decision support tools are developed to aid stakeholders, including policymakers, farmers, healthcare professionals, and environmentalists, in making informed decisions to optimize pest management practices and safeguard health and ecosystems.

*Step 8. Continuous Learning and Adaptation:* ST and SD encourage continuous learning and adaptation. The model is updated regularly with new data, and the analysis is refined to reflect changing conditions and emerging challenges. Stakeholder engagement and knowledge exchange play a vital role in incorporating diverse perspectives and fostering adaptive management practices.

This step-by-step implementation approach offers a comprehensive and adaptive framework for addressing the complex and interconnected challenges of pest management, climate change, and human-animal-environment interactions. It enhances the resilience and sustainability of agricultural systems while safeguarding the health of people, animals, plants, and ecosystems.

### 2.3 Digital twins climate-smart one-health integrated pest management (DT climate-smart OH IPM)

**Fig. 2:**
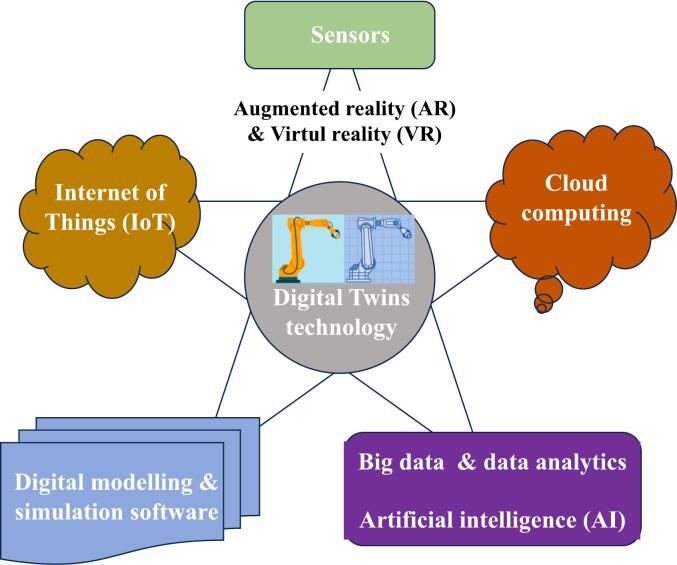
Illustration of the digital twins' fundamental technologies that works synergistically to provide a powerful platform for understanding, analyzing, and optimizing natural systems. The integration of digital twins into operational processes requires robust connectivity and networking technologies. This ensures seamless communication between natural system, IoT devices, data centres, and cloud platforms.

**Fig. 3:**
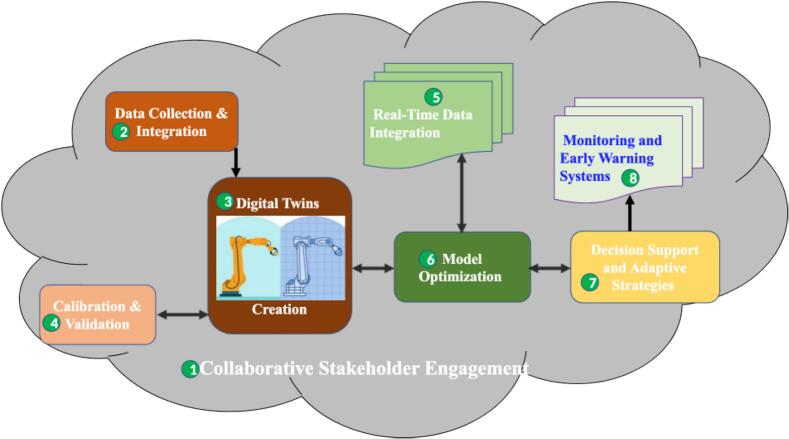
Illustration of the steps involve in the implementation of DT Climate-Smart OH IPM which starts by collecting and integrating data from various sources into a centralized platform to create a DT. The DT is then calibrated and validated using real-world data, continuously updated with real-time information, and optimized for better predictive capabilities. As a decision support tool, it enables stakeholders to explore pest management and health intervention scenarios, guiding adaptive practices. Equipped with monitoring and early warning systems, the DT detects potential pest outbreaks and health risks for prompt action. Collaborative stakeholder engagement is vital throughout the process.

A digital twin (DT) is a virtual replica or simulation of a physical object, process, or system, including agricultural landscapes and pest populations [6,21]. DT IPM pertains to the utilization of digital twins fundamental technologies (Fig. 2) in the realm of pest management. In the context of IPM, DT technology is harnessed to model and simulate pest dynamics, crop growth, environmental conditions, and other relevant factors in a virtual environment. This requires leveraging on real-time data and advanced analytics, to provide a dynamic and accurate representation of pest infestations and their interactions with the environment. This technology offers valuable insights to monitor and predict pest outbreaks, assess the efficacy of pest control strategies, and make informed decisions for efficient pest management. Below is the step-by step implementation of DT Climate-Smart OH IPM (Fig. 3):

*Step 1.* Establish a collaborative network of stakeholders, including farmers, agronomists, technologists, and public health officials, to define the objectives, requirements, and expected outcomes of the digital twin system. Stakeholders provide critical insights into local contexts, practical challenges, and implementation feasibility. Their early involvement ensures the alignment of models and digital systems with real-world needs, improving adoption and effectiveness.

*Step 2.* The second step in implementing DT Climate-Smart OH IPM is to collect and integrate relevant data from multiple sources. This includes data on pest populations, crop health, weather patterns, soil conditions, human health factors, and other environmental variables. The data should be collected from various sensors, satellite imagery, climate databases, health records, and agricultural monitoring systems. Integration of this diverse data into a centralized platform is crucial for a comprehensive analysis.

*Step 3.* Using the integrated data, a digital twin is created to simulate the complex interactions between pest dynamics, climate variables, human health, and the environment. The digital twin should accurately represent the real-world agricultural system and its interdependencies with human and animal health and ecosystem well-being. Sophisticated modelling techniques are employed to ensure the accuracy and fidelity of the digital twin.

*Step 4.* The digital twin is then calibrated and validated using real-world data. Comparison is made between the outputs of the digital twin and observed data from the physical system. Any discrepancies or errors are identified and corrected to improve the accuracy of the simulation. Calibration and validation are iterative processes to fine-tune the digital twin's performance.

*Step 5.* For an effective DT Climate-Smart OH IPM, continuous real-time data integration is essential. The digital twin should be updated with real-time data from weather stations, pest monitoring traps, health databases, and other sources. This ensures that the digital twin remains current and responsive to changing environmental conditions and pest dynamics.

*Step 6.* As the digital twin receives real-time data updates, the underlying models and algorithms need optimization. Model performance is continuously assessed, and adjustments are made to enhance predictive capabilities and accuracy. Model optimization allows the digital twin to provide more reliable insights for decision-making.

*Step 7.* The calibrated and optimized digital twin serves as a valuable decision support tool. It enables stakeholders, including farmers, healthcare professionals, policymakers, and environmentalists, to explore different pest management and health intervention scenarios. The digital twin's simulations help evaluate the effectiveness of various strategies and guide adaptive management practices that respond to changing conditions.

*Step 8:* The digital twin can be equipped with monitoring and early warning systems that continuously track pest dynamics, health indicators, and environmental changes. Alerts and notifications are generated in real-time when potential pest outbreaks, disease outbreaks, or climate-related risks are detected. This enables proactive measures to be taken promptly.

## 3 Results

### 3.1 ST and SD for Climate-Smart OH IPM

**Fig. 4:**
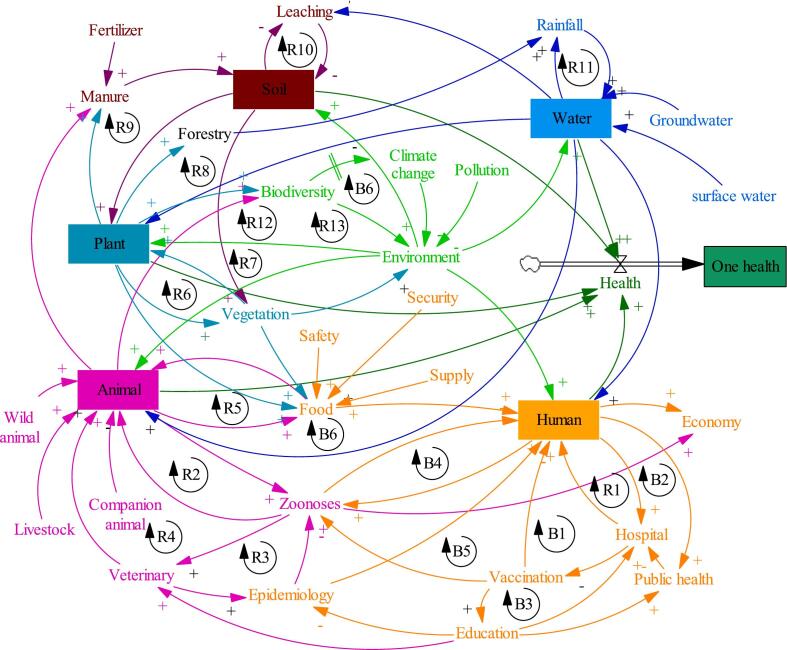
Illustration of the passage explaining the holistic concept of climate smart OH, comprising of five interconnected components (human, animals, plants, soil, and water) and the environment. Understanding the causal links and reinforcing loops between these components is essential for promoting optimal health outcomes in Climate-Smart OH IPM. This approach emphasizes the importance of collaboration and integrated strategies to address health challenges that span across species and ecosystems.

The passage introduces the concept of climate-smart One-OH and its five interconnected components, including animals, humans, plants, soil, and water, within an environmental context (Fig. 4). Climate-smart OH is an approach that recognizes the interdependence of human, animal, soil, water, plant, and environmental health, emphasizing the importance of considering these factors as a whole system. The human component (orange) highlights the significance of human well-being within the larger system, acknowledging the interactions with animals, plants, soil, water, and the environment that influence human health. The animals component (pink) focuses on the health and welfare of both domestic and wild animals, considering the potential for zoonotic diseases and the reciprocal relationship between animal and human health. The plants component (light sea green) underscores the importance of plant health in maintaining environmental balance and supporting human and animal well-being. The soil component (brown) plays a vital role in sustaining plant life and agriculture, thereby affecting food production and human and animal health. The water component (blue) is crucial for supporting life and the health of all living organisms. Furthermore, the environment (light green) encompasses the physical surroundings, ecosystems, and interactions among the five components, providing a backdrop for the entire climate-smart OH system. The causal links, indicated by arrows, illustrate how changes or influences in one component can impact the others. Positive causal links (+) signify factors that increase or decrease together in the same direction, while negative causal links (−) suggest that linked factors change in opposite directions. Additionally, two different loops in the system are introduced: the reinforcing loop (R) and the balancing loop (B). The reinforcing loop indicates that actions or factors lead to growth or decline at an increasing rate, influencing the overall system dynamics. On the other hand, the balancing loop works to limit growth, maintain stability, and achieve equilibrium within the system, ensuring a balanced and resilient ecosystem. This passage provides a comprehensive overview of the climate-smart One-Health concept, emphasizing the interconnectedness and interdependence of human health, animal health, and the environment. Understanding these dynamic relationships and reinforcing loops is crucial for adopting a holistic approach to address complex health and environmental challenges and promote the well-being of all components within the climate-smart One-Health system.

Overall, the interactions between the system variables resulted in 19 feedback loops, consisting of 13-positive (reinforcing: R) and 6-negative (balancing: B) which capture the essential components and variables of the whole system (Fig. 4). Those loops for each component are further described in Table 1. The conceptual system model is then dominated by reinforcing loops suggesting the presence of positive feedback loops within the system. In system dynamics and causal loop diagram analysis, reinforcing loops are represented by loops that amplify changes in a system. This can lead to exponential growth or decline, depending on the nature of the loop. A such system dominated by reinforcing loops can exhibit runaway behaviour. This means that without external intervention or counteracting factors, the system's variables will continue to grow (or decline) without bound. This can lead to situations where the system reaches extreme levels or even collapses if not managed, making OH IPM system more vulnerable to changes. While reinforcing loops can lead to growth and positive outcomes, they can also make a system vulnerable. If there's an unexpected shock or disturbance, a system dominated by reinforcing loops might be less resilient in recovering or adapting to changes.

**Table 1:** Causal loop by system components.

	LOOP DESCRIPTION	IMPLICATION
**Human component**		
R1	Human – Hospital – Human	The increase of human population increases hospital frequentation
B1	Vaccination – Human – Hospital –Human	Vaccination of against diseases increases hospital frequentation
B2	Hospital – Human – Public health – Hospital	Accessibility of people to health facility with good services increase human wellbeing and public health which can decrease the frequency of hospital frequentation
B3	Vaccination – Education – Hospital – Vaccination	Vaccination facilitates education trough healthy of people and through education there is capacity building for hospital services and vaccination
B4	Human – Zoonoses – Human	Human wellbeing is negatively affected by Zoonoses
B5	Zoonoses – Human – Hospital – Vaccination – Zoonoses	Zoonoses weaken the health of people that increase hospital services while vaccination health hospital and reduce zoonoses outbreak

**Animal component**		
R2	Animal – Zoonoses – Animal	Animals are the main reservoir of zoonoses
R3	Veterinary – Epidemiology – Zoonoses – Veterinary	Veterinary through epidemiology should diagnostic zoonoses where the increase will their great concern
R4	Animal – Zoonoses – Veterinary – Animal	As the animals are zoonoses reservoir, they increase the concern of the veterinary, increasing their services for animal treatment
R5	Animal – Food – Animal	Animals are food source
B6	Animal – Food – Human – Zoonoses – Animal	Animals provide food for people while the zoonoses they host affect their health

**Plant, Soil, Water components**		
R6	Plant – Vegetation – Plant	Plants provide vegetation for the nature
R7	Plant – Vegetation – Environment – Plant	Plants are source of vegetation for environment protection that in turn favour plant production
R8	Plant – Forestry – Rainfall – Water – Plant	Plants provide forestry that is source of the rainfall and produce water. Water helps the plant growth.
R9	Plant – Manure – Soil – Plant	The residues from plants produce manure for soil fertility which helps plant growth
R10	Soil – Leaching – Soil	The leaching deteriorates soil structure and texture
R11	Rainfall – Water – Rainfall	The evaporation of water condenses into clouds in the sky which falls as rain

**Environment component**		
R12	Environment – Plant – Biodiversity – Environment	The contribution of plants to the nature biodiversity enhances the quality of the environment
R13	Environment – Animal – Biodiversity – Environment	The contribution of animal to the nature biodiversity enhances the quality of the environment
B6	Environment – Plant – Biodiversity – Climate change – Environment	The contribution of plants to nature biodiversity restores ecosystem against climate change for the environment enhance

**Fig. 5:**
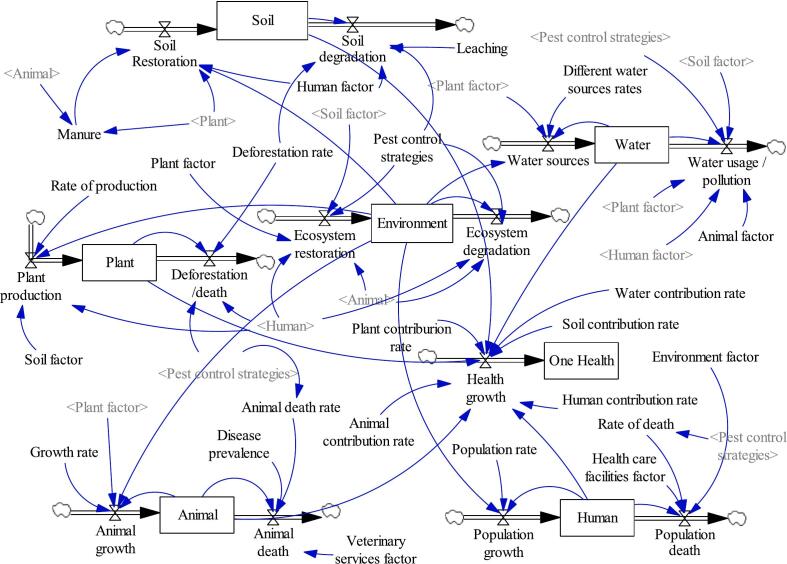
The stocks and flows diagram of the well-being of all components within the climate-smart One-Health system.

The causal loops are transformed into a dynamic model by employing stocks, flows, auxiliary links, and clouds (Fig. 5), which enable the system to be numerically simulated and quantified. Stock and flow diagrams allow for mathematical modelling and simulation. They provide a foundation for creating equations that describe how the variables interact over time, enabling you to perform numerical simulations to observe the dynamic behaviour of the system under different conditions. This diagram is a build computer-based simulations with various scenarios model. This enables to test hypotheses, explore the impact of different policies or interventions, and gain insights into the long-term behaviour of the system before implementing them in the real world. Therefore, stocks and flows diagram helps in understanding the potential outcomes of policy decisions and designing strategies that lead to desired outcomes for the maintenance or improvement of climate-smart One-Health IPM system.

### 3.2 DT climate-smart OH IPM

The use and application of DT approach to for Climate-Smart OH IPM can lead to more sustainable and efficient pest management practices. It empowers farmers and IPM practitioners to make informed decisions, adapt to climate changes, minimize environmental impacts, and consider the broader implications on human and animal health. The specific results would depend on the context, location, and data utilized during the implementation of the approach. Firstly, DT will enhance decision-making by providing real-time and predictive insights, enabling farmers to choose the most suitable pest management strategies tailored to their specific needs and environmental conditions. Secondly, through simulation of various pest management approaches, DT allow for risk assessment, identifying and addressing potential challenges before implementation in the real world. Thirdly, the approach will optimize resource usage, such as water, pesticides, and fertilizers, by pinpointing areas where inputs can be reduced without compromising crop health. Moreover, the climate-smart aspect of DT empowers farmers to adapt pest management practices to changing weather patterns, ensuring their continued effectiveness despite climate fluctuations. Additionally, the use and application of sustainable and climate-resilient pest management strategies in crop production will contribute to mitigating climate change and reducing its carbon footprint. Furthermore, the adoption of OH IPM, promoted through DT, fosters environmentally friendly pest control methods, minimizing reliance on chemical pesticides with detrimental effects on ecosystems and human health. Lastly, considering the interactions between animals, humans, and pests, OH IPM facilitated by DT can play a crucial role in preventing the spread of zoonotic diseases, which are infections that can transfer from animals to humans.

## 4 Discussion

The integration of ST & SD approaches is crucial for developing and implementing effective climate-smart OH IPM strategies. ST allows for the consideration of multiple variables and scenarios simultaneously [22], which is highly relevant in climate-smart OH IPM, where numerous factors need to be holistically assessed, such as climate variables, pest biology, cropping systems, socio-economic aspects, and human and animal health. ST frameworks help map the connections between these variables and evaluate the potential impacts of different interventions and scenarios. In parallel, SD provides a framework to analyse feedback loops and nonlinear dynamics [22] within complex systems, which is essential for understanding the interactions between climate, pests, hosts, control measures, and the environment in climate-smart OH IPM. This allows us to identify optimal interventions for pest management. Furthermore, ST & SD highlight the presence of time delays and lag effects within complex systems [22] which are significant in climate-smart OH IPM due to the delayed responses of pest populations, disease dynamics, and ecosystem resilience to interventions and climate change. The incorporation of time delays into models and analyses, let to anticipate and account for delayed effects in decision-making processes. Addressing trade-offs and unintended consequences is essential in climate-smart OH IPM, as it involves balancing multiple objectives, such as pest control, environmental sustainability, and human and animal health. ST & SD approaches facilitate the exploration of trade-offs and the assessment of unintended consequences associated with different management strategies, enabling the identification of strategies that optimize outcomes across multiple dimensions [23]. Finally, ST & SD frameworks support adaptive management and decision-making under uncertain and changing conditions. In climate-smart OH IPM, these approaches enable iterative learning, continuous monitoring, and adaptive adjustment of strategies in response to new information and emerging challenges [24]. In embracing dynamic and adaptive strategies, we enhance our capacity to respond effectively to dynamic and unpredictable situations in the realm of pest management and climate change impact. Overall, the integration of ST & SD approaches in climate-smart OH IPM empowers us to develop robust and adaptive pest management strategies that consider the complexities of the system and the changing environmental conditions. This approach provides a holistic and forward-looking perspective for promoting sustainable and resilient pest management practices that align with climate-smart principles, ultimately contributing to the well-being of humans, animals, plants, soil, and the environment.

DT Climate-Smart OH IPM represents an innovative and transformative approach that harnesses the power of digital twin technology within the context of IPM. By seamlessly integrating digital twin technology, DT Climate-Smart OH IPM creates virtual models of entire agricultural landscapes, encompassing vital components such as pest populations, crop growth dynamics, and prevailing environmental conditions. This harmonious fusion of virtual modelling with real-time data and advanced analytics empowers DT Climate-Smart OH IPM with a dynamic and highly precise representation of pest infestations and their intricate interactions with the environment. A fundamental advantage of DT Climate-Smart OH IPM lies in its real-time monitoring and predictive capabilities for pest outbreaks. The continuous update of the digital twin model with current data equips stakeholders with valuable insights into pest dynamics, enabling them to proactively anticipate and promptly respond to potential pest challenges. Furthermore, DT Climate-Smart OH IPM serves as a virtual testing ground for evaluating various pest control strategies, offering decision-makers the opportunity to optimize resource allocation by testing and comparing multiple approaches. The simulation of diverse strategies and their potential outcomes within DT Climate-Smart OH IPM empowers stakeholders to identify the most efficient and sustainable options for pest control. When leveraging in this technology, decision-makers can make informed choices that contribute to the safeguarding of agricultural ecosystems and the sustainable management of pest populations. Moreover, DT Climate-Smart OH IPM fosters adaptive pest management practices by continuously updating the digital twin model to reflect changing environmental conditions and evolving pest dynamics in real-time. This adaptive feature empowers stakeholders to adjust their pest management strategies as needed, enhancing their capacity to effectively navigate emerging challenges and uncertainties. DT Climate-Smart OH IPM offers a groundbreaking solution for modern pest management, seamlessly bridging the gap between the physical and digital realms. By harnessing the power of this innovative approach, stakeholders can make well-informed decisions, optimize pest control strategies, and contribute to the sustainable management of pest populations while preserving the health and integrity of agricultural ecosystems.

The combined use of ST, SD, and DT represents a powerful synergy in the domain of climate-smart OH IPM. The integration of these methodological approaches provides a holistic and data-driven foundation for addressing the complexities of pest management challenges in the context of climate change. Through the lens of ST and SD, we can identify feedback loops, leverage points, and tipping points that influence pest dynamics and their impacts on agriculture. Meanwhile, DT enhance our ability to simulate and model pest infestations, crop growth, environmental conditions, and other relevant factors, facilitating dynamic and adaptive pest management strategies. The combination of ST, SD, and DT in climate-smart OH IPM, help to foster a paradigm shift towards a more sustainable and resilient approach to pest management. These methodologies allow us to anticipate and address potential risks, trade-offs, and unintended consequences associated with different pest management strategies. As we navigate the challenges of climate change and its impacts on pest dynamics, the integration of these robust methodologies enables us to optimize resource utilization, enhance agricultural sustainability, and mitigate climate change through environmentally friendly pest management practices. While not primarily focused on zoonotic disease transmission, these strategies indirectly support broader One Health goals by fostering balanced and resilient agroecosystems.

## 5 Conclusion

Embracing ST, SD, and DT in climate-smart OH IPM requires collaboration between various stakeholders, including researchers, agronomists, entomologists, public health officials, policymakers, and local communities. Together, they can co-develop context-specific and adaptive pest management strategies that align with the principles of climate-smart agriculture and OH. The use of these methodological approaches, either individually or combined will pave the way for a more resilient, sustainable, and harmonious coexistence between humans, animals, plants, soil, and the environment, ensuring the well-being of present and future generations.

## CRediT authorship contribution statement

**Henri E.Z. Tonnang:** Conceptualization, Formal analysis, Methodology, Writing – original draft, Writing – review & editing. **Ghislain T. Tepa-Yotto:** Conceptualization, Funding acquisition, Project administration, Writing – original draft, Writing – review & editing. **Bonoukpoè Mawuko Sokame:** Conceptualization, Methodology, Software, Writing – original draft, Writing – review & editing. **Jeannette K. Winsou:** Funding acquisition, Project administration, Writing – original draft, Writing – review & editing. **Manuele Tamò:** Conceptualization, Methodology, Resources, Writing – original draft, Writing – review & editing. **Rousseau F. Djouaka:** Conceptualization, Formal analysis, Funding acquisition, Resources, Supervision, Writing – review & editing.

## Funding

The authors thankfully acknowledge the financial support provided by the International Development Association (IDA) of the World Bank to projects aimed at Accelerating Impacts of CGIAR Climate Research for Africa (P173398, AICCRA-Ghana). IDA helps the world's poorest countries by providing grants and low to zero-interest loans for projects and programs that boost economic growth, reduce poverty, and improve poor people's lives. IDA is one of the largest sources of assistance for the world's 76 poorest countries, 39 of which are in Africa. Annual IDA commitments have averaged about $21 billion over circa 2017–2020, with approximately 61 % going to Africa. This work was partly funded by the Royal Norwegian Embassy in Mali for ‘climate smart agricultural technologies for improved rural livelihoods and food security’ in Mali (Grant MLI-17-0008) and Niger (Grant NER-17-0005).

## Declaration of competing interest

The authors declare that they have no known competing financial interests or personal relationships that could have appeared to influence the work reported in this paper. The funders had no role in the design of the study; in the assemblage and interpretation of data; in the writing of the review paper, or in the decision to publish the results.

## Data Availability

No data was used for the research described in the article.
